# Pathology program directors beliefs on changing answers and literature review

**DOI:** 10.1016/j.acpath.2025.100213

**Published:** 2025-08-13

**Authors:** Gary W. Procop, Ty McCarthy, Paul Jones, Kirk Becker

**Affiliations:** a American Board of Pathology, Tampa, FL, USA; b Pearson Vue, Bloomington, MN, USA

**Keywords:** Answers, Board examination, Changes, Metacognition, Pathology, Performance

## Abstract

The unfounded belief that changing answers during an examination will usually result in a diminished score due to the likelihood of changing correct responses to incorrect responses persists despite overwhelming evidence to the contrary. Ninety two percent of the Pathology Residency Program Directors responding to this survey had been told this sometime during their career. Although the majority of Program Directors reported not propagating this belief, 14 % still reported providing advice to their trainees to not change answers during an examination. A simple, overarching directive concerning changing answers is not possible given the complexity of this topic and the variables that have been discovered that influence answer-changing behavior. The influencing factors include academic ability, item difficulty, preexisting bias, the use of item marking, time for item reconsideration, and the metacognition of the examinees particularly with respect to the confidence they have in the change in the item selection. A more thorough explanation of these influencing factors should be given to trainees prior to an examination to achieve a truer evaluation of their knowledge and skills and improve the validity of the examination.

It has been our experience that many students and trainees, as well as their instructors, have been told not to change answers during an exam because they will more often change a correct answer to an incorrect answer. We were interested in the prevalence of this belief among Program Directors of Accreditation Council for Graduate Medical Education–accredited Pathology Residency Training Programs. We surveyed these Program Directors regarding this concept to understand the prevalence of this belief in our training programs and reviewed the existing literature to provide evidence-based information to educators in our community.

The American Board of Pathology sent a short survey to Pathology Residency Program Directors on January 30, 2025. The survey asked the directors to consider the statement, ‘Be careful about changing your answers on an exam, as it is more likely to change a right answer to a wrong answer.’ The survey then asked each respondent the five questions shown below about the phrase and its subject matter. The survey closed at midnight Pacific Standard Time on February 28, 2025. The questions posed to program directors were as follows:

Question 1: Have you ever heard of this adage or one like it? “Be careful about changing your answers on an exam, as it is more likely to change a right answer to a wrong answer” The answer choices were as follows: Yes or No.

Question 2: Do you believe this adage to be true? The answer choices were as follows: Yes, No, or Don’t know.

Question 3: Did you get this advice from one or more of your mentors? The answer choices were: Yes, No, or I do not recall.

Question 4: Have you given this advice to your graduating residents when they were preparing to take a certification examination. The answer choices were as follows: Yes; If Yes: Why do you give this advice? or No; If No: Why don't you give this advice?

Eighty-seven (87/142; 61 %) Pathology Residency Program Directors responded to the survey. Of these, 92 % (80/87) responded that they had heard the statement or something similar at some point in their careers. When asked whether they believed this statement to be true, a plurality of respondents (48 %, N = 42) stated that they did not know whether the statement was true or not. Of the remaining Program Directors, approximately the same percentage responded that they believed the statement to be true (25 %, N = 22) as those who did not believe the statement to be true (26 %, N = 23).

When asked whether the Program Directors ever received this advice or similar advice from at least one of their mentors, two-thirds of respondents (N = 58) said they have received this advice from a mentor. Twenty-one percent of respondents (N = 18) said they have not received this advice from a mentor, whereas 13 % (N = 11) did not recall.

When asked whether the directors have ever given this advice to their own graduating residents when they are preparing to take a certification examination, most respondents (83 %; 72/87) said they have not given this advice. Of these, 82 % (59/72) provided reasons why they had not given this advice to trainees. Most commonly, 31 % of respondents (N = 18/59) stated that the changing of an answer depended on the situation. Specifically, if an examinee remembered something important about the content of the question later in the exam, then they probably should change the answer. Conversely, the respondents advised against changing answers if the examinee remained unsure of the answer. Twenty-seven percent of these respondents (16/59) simply stated that they either believed the advice was not correct or they were simply not sure. Twenty percent of respondents (N = 12/59) said they had not seen data or the literature supporting the statement.

Seventeen percent (15/87) of Program Directors responded that they have given the stated advice. The three common themes that arose from their responses were as follows: 1) trusting instincts is best especially when certainty is low (40 %, 6/15), 2) there are tendencies to overthink the question when one is nervous (33 %, 5/15), and 3) personal/anecdotal experience in these situations (27 %, 4/15).

The supposition that changing answers will more likely negatively impact the test taker (i.e. they are more likely to change a correct response to an incorrect response) dates back at least to 1929.^1^^,^^2^ This idea has permeated the fields of education, liberal arts and sciences.^3^ Benjamin et al. reported that 55.2 % of faculty believed that changing answers on an assessment would lead to a poorer outcome for the student and this notion continues to persist.^4^^,^^5^ Our survey of Pathology Program Directors disclosed that the vast majority of Program Directors (i.e. 83 %) had been told this sometime during their career and there was a persistent lack of clarity concerning whether or not the changing of an answer on an examination would more likely diminish the overall score. About half of the respondents did not know if this was correct, whereas the other half were split between believing it was true versus believing it was false. Fortunately, the vast majority of Program Directors reported that they do not propagate this concept to their trainees.

Overall, most studies in this area have demonstrated that students usually benefit from changing answers (i.e. incorrect-to-correct changes are more often made). For example, Johnson reported a small study of 102 students wherein more students made an incorrect-to-correct change (71 students) than a correct-to-incorrect change (31 students) (P < 0.005) after subtracting out students who made an equal number of incorrect-to-correct and correct-to-incorrect answer changes (3 students) and those who made no changes (32 students).^6^ Similar findings have been reported by a number of other educational researchers.^7^, ^8^, ^9^, ^10^, ^11^ A meta-analysis of 75 studies on answer changing demonstrated that 57 % of answer changes were from incorrect-to-correct, whereas 21 % answer changes were from correct-to-incorrect.^12^ Although these studies serve to debunk the blanket directive to not change answers on an examination because the participant will more likely change a correct response to an incorrect selection, a variety of caveats exist. The evidence suggests that the likelihood of changing a correct-to-incorrect answer and vice versa is multifactorial, but influencing variables are understood and could be used by the metacognitive examinee to determine if they should consider changing an answer.

Bias due to the instillation of the belief that changing an answer once it is recorded is more likely to result in a correct-to-incorrect change may influence the overall outcome for the participant. If a student has a preexisting belief that changing an answer on an exam is more likely to be detrimental to their overall score, they may not change an item response even if they feel that another response is correct. Bias against answer changing may be driven by inaccurate advice given to students by their instructors or mentors, which may in turn decrease the number and types of test-taking strategies that are available to them.^13^

There are conflicting reports regarding the correlation between academic ability and answer-changing behavior with some studies demonstrating a significant negative relationship and others reporting nonsignificant differences.^3^ Crabtree et al. reported that the highest performing students logged the fewest answer changes and when an answer change was made it was more likely a single incorrect-to-correct change.^14^ In contrast, the lower performing students logged the most answer changes, had a tendency to make multiple changes to an item, and more often ended in an incorrect final selection.^14^ These findings suggest that superior academic preparation will benefit the participant in both knowing the correct answer initially (i.e. not making an answer change), as well as making the correct answer change should one be needed.

Item difficulty is another variable that influences answer changing. While investigating the impact of item difficulty and answer changing, Best reported that overall more students increased their test scores by changing answers (i.e. an incorrect-to-correct change; N = 1031) compared with those who lowered their scores (i.e. a correct-to-incorrect change; N = 348).^15^ Not surprisingly, the answers to items categorized as difficult, based on the item performance compared with the median percent correct in the examination, were more frequently changed compared with items that were similarly categorized as easy.^15^ Individuals in all tertiles of the class (i.e. top, middle, and lower) changed the answers to difficult items more frequently compared with easy items. Further analysis disclosed that there was a tendency for more students in the top and middle tiers to make more incorrect-to-correct changes on the difficult items, whereas students in the lower tier made more incorrect-to-correct changes on the easy items. This further underscores the importance of adequate academic preparation; the better students had to ‘think through’ items that were difficult, but not the easy items, whereas poorer performing students even had to ‘think through’ the easy items. The examinees were less likely to benefit from reviewing items if the content was above their ability level; in such instances, there was little improvement in their test scores if answers were changed.^16^

The amount of time that the examinee is taking to make the decision to change the response to an item provides a clue to the examinee regarding the likely efficacy of their decision. When Crabtree et al. studied answer changing, they found that the average time to answer a question that was not changed was 51 s, which was similar to the time it took a student to make a single incorrect-to-correct change (i.e., 45 s). In stark contrast, the times it took for students to make a single correct-to-incorrect change (2 min, 3 s) or an incorrect-to-incorrect change (i.e. 2 min, 2 s) were significantly longer (P < 0.001), suggesting that the students were struggling to determine the correct response (i.e. they were not confident in the change).^14^ Interestingly, this timing difference was not retained if there were multiple changes to an item. These researchers warn that revisiting the responses to items that require extended periods of contemplation for a change is not always a beneficial testing strategy and could result in a negative outcome.^14^

More recently, the role of metacognition in the effectiveness of answer changing has been investigated.^13^^,^^16^ Metacognition, in this instance, refers to the ability of the individual to monitor and regulate their behavior when taking an examination. Metacognition includes conscious cognitive and affective experiences that may include feelings of confidence and judgment that enhance test-taking decisions.^16^ Interestingly, Swanson reported that examinees with low aptitude but high metacognitive skills performed better than examinees with higher aptitude but lower metacognitive skills.^17^ Some educational researchers contend that individuals with a high level of metacognition who engage in self-monitoring are more capable of selecting test-taking strategies that will result in an enhanced score.^18^ Monitoring is the ability to assess strategy while engaged in learning and is an important metacognitive strategy for the control of cognition while taking a test.^19^ This is distinctly different from what has been termed ‘test-wiseness,’ wherein the examinee uses the characteristics and format of the exam or testing situation to achieve a higher score.^20^ When Kitsantas studied monitoring, she found that the students who reported significantly more monitoring were the high scorers compared with the lowest scorers.^21^

One of the tools used in monitoring is the ‘marking’ of items for which the participant is unsure of the answer so that they may return, if time remains, and consider those items further. The marking of difficult items for later consideration is a commonly used strategy that has been found to be used more frequently by high achievers as opposed to very low achievers.^22^, ^23^, ^24^ It has been proposed that marking may serve as a mechanism to facilitate retrieval of information from long-term memory.^25^ Not surprisingly, Papanastasiou et al. demonstrated that students with a higher level of test-taking metacognition were more likely to employ item marking strategies (*P* < 0.001) and, in-turn, those who used item marking strategies were more likely to change answers than those who did not (*P* < 0.001); importantly, the students who reported changing answers more frequently also had higher test scores (*P* < 0.001).^13^

Confidence in a response is a function of metacognition (i.e. the candidate knows that they know the correct answer). The hypothesis that the confidence that a student has in a particular answer may be important in the examinee’s willingness to change the answer was first investigated in 1983 by Skinner, who reported a high probability (i.e. 74.6 %) of an incorrect-to-correct answer change, if the student was confident of making this change.^26^ Stankov showed that confidence in the correctness of a test answer was the best concurrent predictor of achievement^27^

In conclusion, there is no simple answer to the question regarding the efficacy of changing answers. The evidence is clear, however, that a simple blanket warning to students not to change their answers during an examination is not only incorrect, based on numerous studies, but likely harmful due to the introduction of bias that can disadvantage the examinee. Substantial evidence exists that changing answers actually improves overall scores but there are caveats and subtleties to consider. These include the academic preparedness of the individual (i.e. smarter is always better), item difficulty, bias, the metacognition (e.g. confidence in the answer change) and test-taking strategies (e.g. marking of items) of the individual taking the examination. If an individual is confident that they have marked an item incorrectly, which is a decision that usually occurs quickly (i.e. without extended contemplation), then they should not hesitate to change the response to that which they believe is correct.

## Funding

None.

## Declaration of competing interest

None.
